# Giant Folliculosebaceous Cystic Hamartoma of the Face

**DOI:** 10.3390/dermatopathology11010004

**Published:** 2023-12-31

**Authors:** Ramona Tasar, Melanie Peckruhn, Jörg Tittelbach

**Affiliations:** Department of Dermatology, Jena University Hospital, Friedrich Schiller University Jena, 07747 Jena, Germany

**Keywords:** giant folliculosebaceous cystic hamartoma, hamartomas, adnexal tumors, sebaceous glands

## Abstract

Folliculosebaceous cystic hamartoma (FSCH) is a rare and benign form of cutaneous hamartomas. These skin lesions often lead to clinical and histopathological misdiagnosis due to their similarities to cutaneous lesions with overproduction of clustered sebaceous glands. Clinically, the lesions often present as solitary, skin-colored, pedunculated warts to cauliflower-like, exophytic papules and nodules, usually with a diameter ranging 0.5–1.5 cm that rarely exceed 2 cm in size. Only a small number of giant variants are reported in the literature with a diameter in the range of 5–23 cm. The vast majority of the lesions appear in the central face and show a striking predilection for the nose, ears, and scalp, but also emerge on the nipples, extremities, and genitals. Histologically, the epithelial components of folliculosebaceous cystic hamartoma comprise dilated infundibular cystic proliferation with surrounding mesenchymal components, which commonly include fibroplasia and vascular and adipose tissue proliferation. These histological characteristics were coined by Kimura and colleagues (1991). To the best of our knowledge, our case represents the biggest variant of giant folliculosebaceous cystic hamartoma.

## 1. Introduction

Hamartomas are benign lesions composed of aberrant, disorganized growth of mature tissues indigenous to the anatomic area of occurrence [1]. Folliculosebaceous cystic hamartoma (FSCH) is a rare subtype of cutaneous hamartoma, and the first five cases were first characterized by Kimura et al. in 1991 [2]. The age of onset for FSCH is variable but often begins in early childhood [3,4]. FSCH has a striking predilection for the scalp and central face, with a tendency to occur around the nose [2,5,6,7], with the vast majority of lesions presenting as 0.5–1.5 cm exophytic, solitary, flesh-colored, asymptomatic, rubbery-to-firm, partly umbilicated nodules and papules [8]. Clinically, there are no distinctive features, and the diagnosis of FSCH is made coincidentally, based on the histology, after clinicians suspected similar diagnoses such as intradermal nevus, nevus lipomatosis superficialis, sebaceous hyperplasia trichofolliculoma, dermoid cyst, or soft tissue neoplasm. Histopathologically, it is composed of an infundibulo-cystic structure with radiating sebaceous glands, laminated fibroplasia encircling the epithelial component, mesenchymal elements, blood vessels, a variable proportion of adipose tissue, clefts between the epithelial component, and confinement to the dermis [2,9]. Most reported cases of FSCH did not exceed 25 mm in diameter; only nine cases of a giant variant have been reported so far [3,4,10,11,12,13,14]. There is no clear definition for the term “giant”, but in previous publications, a diameter of 5.0 cm or larger is classified as giant and occurs in different anatomical regions, including the back, vagina, nipple, axilla, the upper arm, and the shoulder [3,4,10,12,13,14,15]. In most cases, the tumor appears as a solitary lesion, but in rare cases, multiple lesions may also occur [16]. These tumors show slow growth and can be asymptomatic for decades, and minor complaints have generally been recorded, such as pruritus, discomfort, a tense sensation, or pressure pain [8]. We report a case of a giant follicuclosebaceous cystic hamartoma of the face, which, due to its size, led to a drooping lower eyelid and several complications.

## 2. Report of a Case

An 84-year-old male patient presented with a tumorous flesh-colored, partially erythematous skin lesion of the left side of the face, which initially appeared as a solitary patch on the left cheek 62 years ago. This lesion had constantly increased in size proportional to body growth until the entire left side of the face was affected. At the time of his first presentation in our dermatological department, the lesion comprised, in addition to structures in the face, parts of the head, ear, and neck. Anamnestically, his family history was negative for skin diseases, and his children were born healthy, and he has further healthy grandchildren.

**Figure 1:**
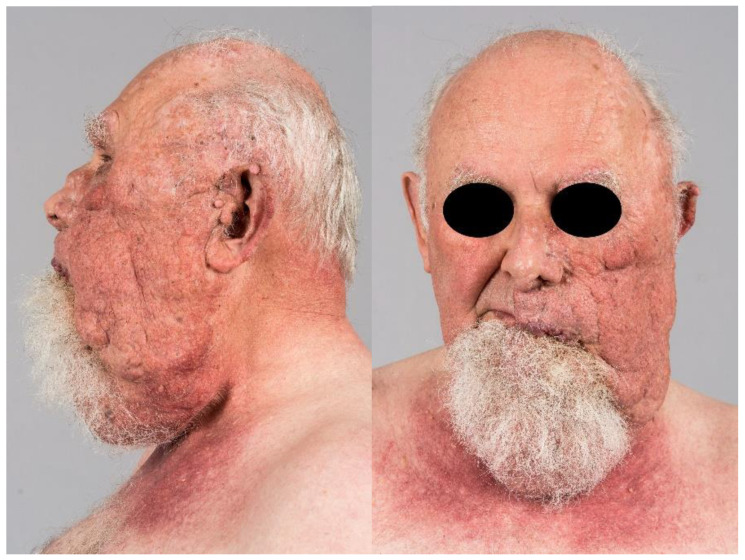
The skin lesion is a flesh-colored, cerebriform, nodular mass, 26.0 × 11.0 cm, on the left side of the face. The smooth surface showed up papillomatous with enlarged follicle-like pores and a waxy discharge. The neck showed a pendolous, dewlap-like mass.

On examination, the skin lesion—surrounding the entire left side of the face (Figure 1), parts of the head, ear, and neck—presented as a flesh-colored, partially erythematous-livid, cerebriform, nodular mass with blurred borders to the adjacent healthy skin and reached approximately 26.0 × 11.0 cm in size. The left half of the neck showed a pendulous, dewlap-like mass. The surface appeared papillomatous with enlarged follicle-like pores. Due to the constant growth of the skin lesion, the patient underwent two cosmetic surgeries in 1991 and 1992, as the weight affected the left eyelid and pulled it downwards. In addition, the patient reported wax-like secretions that could be expressed from the umbilicated center of the papules. He has been taking Isotretinoin in different dosages since 1993.

**Figure 2:**
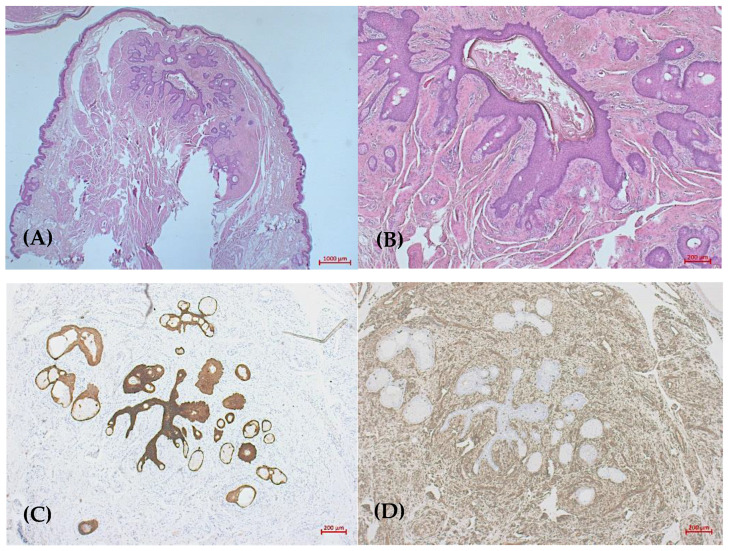
(**A**) Dense mesenchymal component surrounding epithelial cystic structures (HandE, ×12.5); (**B**) smaller mature sebaceous gland lobules and dilated follicular structure (HandE, ×50); (**C**) pancytokeratin staining shows epithelial component (MNF-Kon, ×50); (**D**) surrounding fibrotic connective tissue (Vimentin, ×50).

Differentially, we discussed segmental neurofibromatosis, nevus sebaceous type Schimmelpenning–Feuerstein–Mims syndrome, cervicofacial actinomycosis, and late-stage rosacea glandular hyperplastic type III with massive phyma formation. Histopathological examination demonstrated in hematoxylin-eosin-stained sections a polypous tumor consisting of a cystic epithelial lesion with dilated follicular structures, localized in the dermis, surrounding a prominent mesenchymal component and irregular sebaceous glands (Figure 2A). The epidermis is hyperkeratotic and acanthotic. More deeply, the lesion showed central dilated follicular structure and smaller mature sebaceous gland lobules radiating into the surrounding area, and the cystic cavity had infundibular keratinization (Figure 2B). The epithelial component was positive for pancytokeratin (MNF-Kon) (Figure 2C). The stroma surrounding the epithelial units consisted of dense fibrocytic connective tissue, which was positive on vimentin staining (Figure 2D). Immunohistochemically, within the stroma, there were numerous smaller vascular structures that showed immunoreactivity to CD31, CD34, and SMA staining; outside the vascular structures, no increased positivity was shown in these stains. The stromal cells were negative for Desmin and S100.

In addition, we performed CT of the skull and MRI of the head and neck soft tissues to exclude possible osseous involvement or infiltration of vascular or nerve branches.

In the examinations, there was no osseous involvement. There was sclerosis of the skull calotte of the temporal bone with compression of the adjacent cerebral hemisphere without evidence of edema. These findings were considered to be age-related. Furthermore, there was a nasal septum deviation to the right and an atypical configuration of the medial nasal conchae. Overall, there was no further action required.

Because surgical excision of the giant FCSH was undesirable due to its size, poor demarcation, and hemifacial involvement, we continued the treatment with Isotretinoin 20 mg/day.

## 3. Discussion

Since Kimura et al. described the first five cases of FSCH in 1991, numerous cases of FSCH have been published over the years, but only a few have been described as giant FSCHs. The incidence of FSCH is controversial. FSCH is described as a very rare condition. However, there are more cases than believed, with Wu et al. reporting 14 cases among 15,000 archived tissue samples (0.09%) [17]. Furthermore, there are studies with 153 cases of FSCH from 251,309 samples (0.06%) in today’s literature [8]. Although there are irregular publications, this type of tumor is not exceedingly rare. Strikingly, more than 80% of all cases were published in East Asia, which may provide information that the incidence of FSCH is higher in the Asian region than in Europe.

Since the first clinical and histological features were proposed by Kimura et al. in 1991, they have not changed significantly over the years. Albrecht introduced the term hamartoma to describe a tangle of indigenous tissue [18]. The term “hamartoma” describes a benign, localized malformation of cells that resembles neoplasm, essentially due to an overgrowth of multiple abnormal cells [19]. Based on this term, folliculosebaceous cystic hamartomas define a mixture of epidermal, dermal, adnexal, and hypodermal cells that show histologically dilated follicular structures with infundibular cystic structures and surrounding multiple sebaceous lobules. [2,8,9,16,20,21]. In the current literature, only eight cases were reported with giant folliculosebaceous cystic hamartoma. There is no clear definition for the term “giant”, but it might be classified as having a diameter of 5.0 cm or larger [3,4,10,12,13,14,15,22]. Two of these are located on the upper extremities, more precisely on the right forearm and the left upper arm, ranging in size from 13.0 to 15.0 cm [13,22]. Two further cases have been described in the genital areas, with a diameter of 5.0 cm of the vulva and up to 23.0 cm of the scrotal region [10,14]. In other cases, the giant lesions presented on the right cheek with a diameter of 10.0 × 8.0 cm and on the upper back with a size of 7.0 × 3.0 cm [12,14]. Moreover, two congenital cases of giant variants have been described on the neck and on the thigh [3,4]. The majority of the reported giant FSCH cases had been present since birth, or at least since early childhood, with a possible late visit to the clinics. The clinical characteristics of our patient were similar to the previously reported cases of giant FSCH. Our patient developed the lesion in his early childhood, with a very slow progression of growth in the past 80 years. In most reported cases, the giant FSCH was surgically removed due to favorable localizations and sharp dermacations. On the contrary, surgical intervention in our case was contraindicated due to the involvement of critical structures and the advanced age of the patient at the time of presentation. Instead, Isotretinoin 20 mg/d was started years before, probably due to the inhibitory properties of retinoids on sebaceous gland activity [23]. Interestingly, two of the giant variants were treated with Isotretinoin or Acitretin [10,12]. In the case of Isotretinoin, the lesion returned to full size after cessation of the drug, and surgical excision was recommended [12]. In the case of Acitretin, prior to the intake, the tumor was removed by layered CO2 laser ablation and did not return in the next 18 months of observation [10].

The histologic criteria used for the diagnosis were developed by Kimura et al. The authors proposed five specific histologic features for the diagnosis of folliculosebaceous cystic hamartoma: (I) an dilated infundibular cystic structure with bundles of radiating sebaceous lobules; (II) a compact fibroplasia around the epithelial component; (III) surrounding mesenchymal changes including dense collagen tissue, adipocytes, and an increased number of small venules; (IV) clefts between the altered stroma and the adjacent compressed fibrous tissue; and (V) additional stromal changes with confinement of the process primarily to the dermis [2].

Compared to the other described giant variants, the individual clinical presentation might vary, but histologically, the previously reported cases showed cystic structures with peripheral radiation of dilated follicular structures, surrounded by mesenchymal components and irregular or malformed mature sebaceous glands. The cystic cavity shows typical infundibular keratinization. The surrounding stroma consisted, depending on the localization, of dense fibrocytic connective tissue or fibroadipose tissue [3,4,10,12,13,14]. In the reported case, the histological features were highly similar to those of the other described cases of giant folliculosebaceous cystic hamartoma [3,4,10,12,13,14,15].

A variety of differential diagnoses should be considered if some of these parameters match histologically. They include sebaceous hyperplasia, sebaceous trichofolliculoma, fibrofolliculoma, or dermoid cyst, just to mention a few. Sebaceous hyperplasia is similar to FSCH dilated follicular structures with associated sebaceous elements but is more superficially located in the dermis, and the dilated follicular structures are directly connected with the epidermal surface [7]. A dermoid cyst shows folliculosebaceous elements but is a true cyst, is frequently located in subcutaneous fat, and is possibly connected with other adnexal structures (eccrine and apocrine glands) [7]. A fibrofolliculoma shows similar characteristics to a folliculosebaceous cystic hamartoma: a dilated follicular structure filled with keratin but lacking the sebaceous component of FSCH [24]. Beyond that, histologically, a fibrofolliculoma displays thin, elongate stands of infundibular epithelium (mantle-like) extending from the cystic area [2]. One of the most controversial differential diagnoses in the literature is sebaceous trichofolliculoma (ST). There are numerous authors who believe that ST and FSCH are the same entity [25,26]. For example, in 1998, Schulz and Hartschuh claimed that FSCH represents a late phase of ST in its differentiation. They reported that ST consists of pathological cyclic hair follicle changes and assumed that FSCH is a very late stage of ST because of the subsequent replacement of the secondary follicles by the developed sebaceous elements in association with stromal enlargement [26]. However, this statement has already been refuted by many authors and has not been universally accepted [4,9,17,20,27]. Although ST and FSCH have many histological features in common, ST lacks mesenchymal elements and, in contrast to FSCH, has a secondary follicle [17]. Moreover, sebaceous trichofolliculoma shows rudimentary follicles connecting to the infundibular cystic wall and adjacent adnexal structures and usually presents within the follicular structures, and a mesenchymal component is said to be absent [7,8]; however, it is more superficially located. Thus, the histological evidence of both secondary follicle and prominent sebaceous gland hyperplasia should be diagnosed as ST rather than FSCH [28]. In addition, Misago et al. analyzed 40 cases of ST, in which the lesions were staged in three chronological stages: the early, advanced, and late stages, respectively. They observed no replacement of secondary follicles with sebaceous elements, so that FSCH and ST are two distinct entities [20,29].

We further considered two potential differential diagnoses. One of them is called Schimmelpenning–Feuerstein–Mims syndrome (SFMS), which is a congenital neuroectodermal symptom complex with nevus sebaceous, malformations, and the possibility of developing dysplasia of the eyes, skin, brain, skeleton, and heart [30]. Furthermore, this syndrome is often combined with hypophosphatemia. Our patient showed no extracutaneous, in particular, neurological symptoms. The common feature of FSCH and SFMS is the ipsilateral expression of the lesion.

Likewise, the suspicion of cervicofacial actinomycosis was excluded after the histologic evaluation due to the absence of sulfur granules and clusters of granulomatous tissue [31].

In summary of the represented cases up to the present date, the typical clinical presentation of FSCH appears in the form of solitary, unilateral, skin-colored, protruding/pedunculated benign papules or nodules, which occur commonly on the face, especially on the nose [8,21,27,32]. Outside of facial predilection, other cases have already been described on the auricle and scalp [24,33,34,35], lower extremity [36], genital [11,37,38], nipple [16,39,40], upper lip and labial mucosa [41], and back [42]. Our case illustrates the constantly growing nature of FSCH, as evidenced by the advanced age of our patient at the initial diagnosis. This consistently growing character led to the classification of a “giant” variant in eight current cases. In addition, our case provided insight into the benign nature of the lesion; however, the size of the lesion can affect the surrounding structures, as in our case, the extent led to an ectropion of the left eye and consequently two surgical interventions. Our case provides room to speculate whether the size of the lesion described can be explained, at least in part, by the location and advanced age of the patient.

To the best of our knowledge, this is the ninth and largest case of giant folliculosebaceous hamartoma. Compared with the histopathology of the previous literature, we found concordances. Thus, we were able to make a specific diagnosis.

## Data Availability

The data presented in this study are available on request from the corresponding author.
